# Downstream Effects of a Hydroelectric Reservoir on Aquatic Plant Assemblages

**DOI:** 10.1100/tsw.2002.142

**Published:** 2002-03-16

**Authors:** Ivan Bernez, Jacques Haury, Maria Teresa Ferreira

**Affiliations:** Forestry Department, I.S.A.-U.T.L., Tapada da Ajuda, 1349-017 Lisboa Codex, Portugal; U.M.R. E.Q.H.C., E.N.S.A.R.-I.N.R.A., 65 rue de Saint Brieuc, 35 042 Rennes Cedex, France

**Keywords:** river, macrophytes, hydroelectric dam, impoundment, regulated flow, macroalgae, hydrophytes, emerged rhizophytes, intermediate disturbance hypothesis, Brittany, ecohistorical approach, river basin history, historical changes

## Abstract

Macrophytes were studied downstream of the Rophémel hydroelectric dam on the River Rance (Côtes d’Armor Department, western France) to assess the effects of hydroelectric functioning on river macrophyte communities. We studied ten representative sections of the hydro-peaking channel on five occasions in 1995 and 1996, on a 15-km stretch of river. Floristic surveys were carried out on sections 50 m in length, and genera of macroalgae, species of bryophyta, hydrophytes, and emergent rhizophytes were identified. For the aquatic bryophytes and spermatophytes section of our study, we compared our results with 19^th^ century floristic surveys, before the dam was built. During the vegetative growth period, the hydro-peaking frequency was low. The plant richness was highest near the dam. The macrophyte communities were highly modified according to the distance to the dam. The frequency and magnitude of hydro-peaking was related to the aquatic macrophyte richness in an Intermediate Disturbance Hypothesis position. However, the results of the eco-historical comparison with 19^th^ century floristic surveys point to the original nature of the flora found at the site. Some floral patterns, seen during both periods and at an interval of 133 years, were indicative of the ubiquity of the aquatic flora and of the plants’ adaptability. This demonstrates the importance of taking river basin history into account in such biological surveys.

### INTRODUCTION

River macrophytes have been used for river water quality assessments, but are still not commonly used as subjects of river ecology research[1]. Moreover, only a few studies have investigated specific stretches of rivers downstream from dams, though many downstream effects have been described[2]. The downstream effects of water regulation on biota and ecosystems have been studied in relation to large hydroelectric dams[3], and in these particular reaches, most of the studies on plants focussed on riparian vegetation[4,5,6]. There is little knowledge in France about the effects of hydroelectric dams on macrophytes, except for some specific studies on biomass[7,8] and periphyton[9]. Aquatic macrophytes are viewed as an engineering management problem, and many macrophyte studies look at weed control problems in or below[10] the dams.

Previous studies have shown that dams change the physical and biological parameters[11] of aquatic environments. Comparisons of floral inventories before and after the regulation of water flow are deemed to be the best way of assessing the effects of a dam[12], although historical data are scarce, and their use must be subject to certain methodological precautions[13]. Downstream effects of hydropower impoundment on flora include changes to community structure[14,15]. The non-natural habitats generated by flow disturbance create prime areas for opportunist species, which expand rapidly and modify community composition[16].

A previous study at the river scale showed the highest richness to be downstream from the Rophémel dam on the river Rance[17]. In the present study, we focus on the distribution of macrophytes downstream from this dam on a gradient of distance to assess the effects of an impoundment of the river Rance on aquatic macrophytes. We set out to understand how the impoundment modified the richness and the distribution of river macrophytes. We discuss the relative influence of the dam discharges and of the characteristics of that specific reach of river. We also compare the present floral composition with 19$^{th}$ century studies.

### METHODS

#### General Features and Flow Regulation

The Rophémel dam was built in 1938 and is located on the river Rance (Fig. 1). The Rance is in Northern Brittany, western France, flowing into the English Channel between Dinard and St-Malo. Mainly underlaid by siliceous rocks (e.g., granites), the Rance crosses the limestone “mer des Faluns” downstream from the hydroelectric Rophémel dam. Rophémel is a reservoir holding 5 million m$^3$ of water, with an area of 80 ha and a maximum depth of 23 m. The flow and frequency of hydro-peaking events for 1984 to 1994 are shown in Fig. 2, with a monthly average flow of 1.91 m$^3$ s$^{-1}$ during that 10-year period. The maximum outflow for this period was 58 m$^3$ s$^{-1}$, in February 1988, and minima occurred very frequently during each summer’s vegetative growth periods (the minimal flow requirement is fixed at 0.1 m$^3$ s$^{-1}$). During the floral studies in 1995 and 1996, there was a monthly average flow of 1.90 m$^3$ s$^{-1}$.

Downstream from the dam in the hydro-peaking channel, we selected ten sections in five stretches of river on a gradient of distance (A: <0.1 km; B: 0.1 to 1 km; C: 1 to 5 km; D: 5 to 10 km; E: 10 to 15 km; Fig. 1).

#### Macrophyte Field Studies

The floristic surveys consisted of estimating the percentage of cover for each plant on a 50-m long section[18]. The survey was carried out in May to June, July, and September 1995, and in

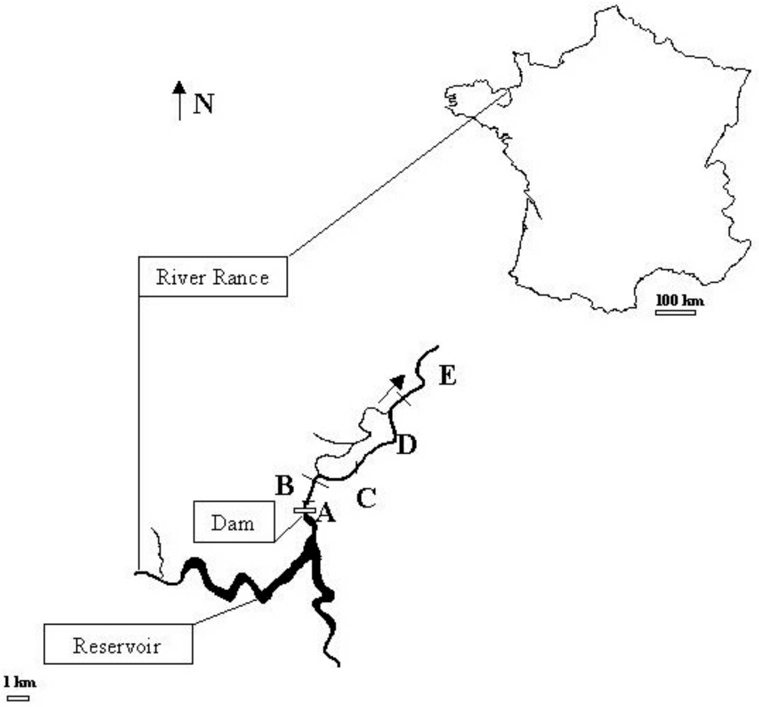

**FIGURE 1.** Localisation of the regulated river Rance in Western France downstream from the reservoir of Rophémel. The studied stretches of the hydro-peaking channel are located on a gradient of distance from the dam (A: <0.1 km; B: 0.1 to 1 km; C: 1 to 5 km; D: 5 to 10 km; E: 10 to 15 km). The arrow shows the direction of the current.

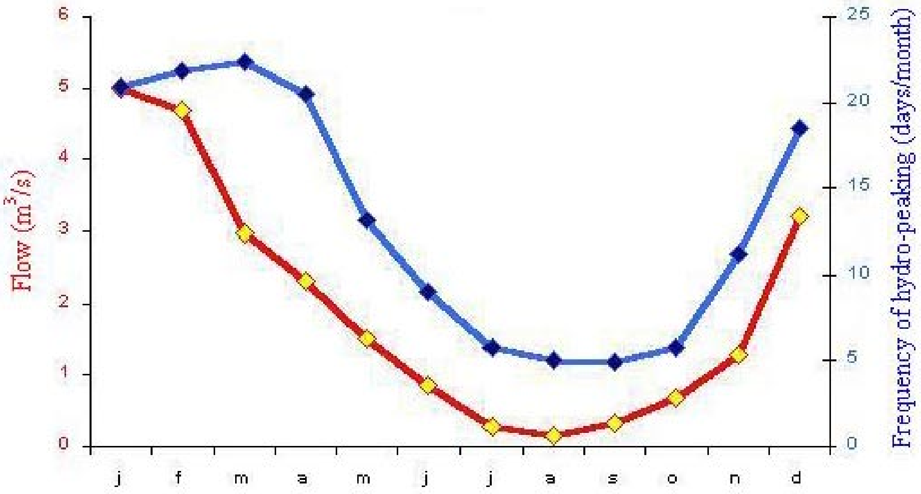

**FIGURE 2.** The mean of the monthly hydro-peaking frequency in number of days per month when hydro-peaking events occurred and out-flow values of these hydro-peaking events in the 10-year period before the floristic study.

May to June and July 1996, on two 50-m sections for each of the five stretches of river, A to E, downstream from the dam. The same surveyor estimated species cover. Macrophytes included species of spermatophytes (taxonomy from Tutin et al.[19]), species of bryophytes (taxonomy from Smith[20,21]), and genera of algae (taxonomy from Bourrelly[22]).

### Eco-Historical Comparison

Historical plant references included three different reference sources on two taxonomic groups (spermatophytes[23,24] and aquatic bryophytes[25]). The bulk of the information was obtained from Mabille[23], whose sampling activity took place between 1861 and 1865 in the Rance River valley. Lloyd's references[24] were used for the description of the taxa, and we consulted his herbarium. Camus' work[25] completed the study with an interesting replica involving another taxonomic group. At the river scale, former work[26] pointed to the similarity between the two lists at an interval of 133 years. In our present survey, we focused on the actual downstream channel of the hydroelectric dam, and we compared the lists before and after the dam's construction. We extracted from the 19$^{th}$ century literature the data collected in the part of river corresponding to the actual hydro-peaking channel itself. Only carefully qualitative comparisons were done, due to the differences in methodology and objective[13,27].

### Data Analysis

The data set consisted of 50 samples (corresponding to two sections for five stretches on five dated occasions). We compared the floristic richness both for the section (50 m) and for the stretch, using a nonparametric test (Wilcoxon)[28,29] and SYSTAT 9.0.[30]. A correspondence analysis (CA) using the CANOCO 4.0 programme[31] was carried out to study the downstream effects by comparing plant species composition and distribution along the channelled river. To improve the ordination trends, rare species were downweighted.

## RESULTS

### Levels of Hydro-Peaking Events

The level of hydraulic perturbation in the 10 years prior to our study was connected to the hydro-peaking frequency, expressed as the number of days in a month during which a hydro-peaking event was recorded, and to the mean flow of the hydro-peaking events during each month (Fig. 2). Hydraulic perturbation decreased, with both lesser frequency and magnitude, when the vegetative growth period started (March to April), and increased when it finished (October). During the summer period, hydro-peaking events were scarce (mainly ecological flow requirements) and presented low levels.

### Vegetation Patterns Downstream From the Dam

Correspondence analysis is commonly used by ecologists to summarise and analyse biological data on species distribution[32]. The first axes are interpreted as latent variables and are presumed to relate to underlying variables. The results of the correspondence analysis applied to the entire vegetation data set are shown in Fig. 3a and 3b, which set out the main features of vegetative spatial patterns.

The eigenvalues of the first two axes were 0.22 and 0.12, respectively, and together they accounted for 27.4% of the total variance on the species data. This level of eigenvalues is commonly obtained in community ecology studies[31]. The respective floral lists collected on the sites during the 2 years drew a very clear distinction between the different reaches of river, with the exception of the two situated more than 5 km away from the dam (stretches D: 5 to 10 km and E: 10 to 15 km).

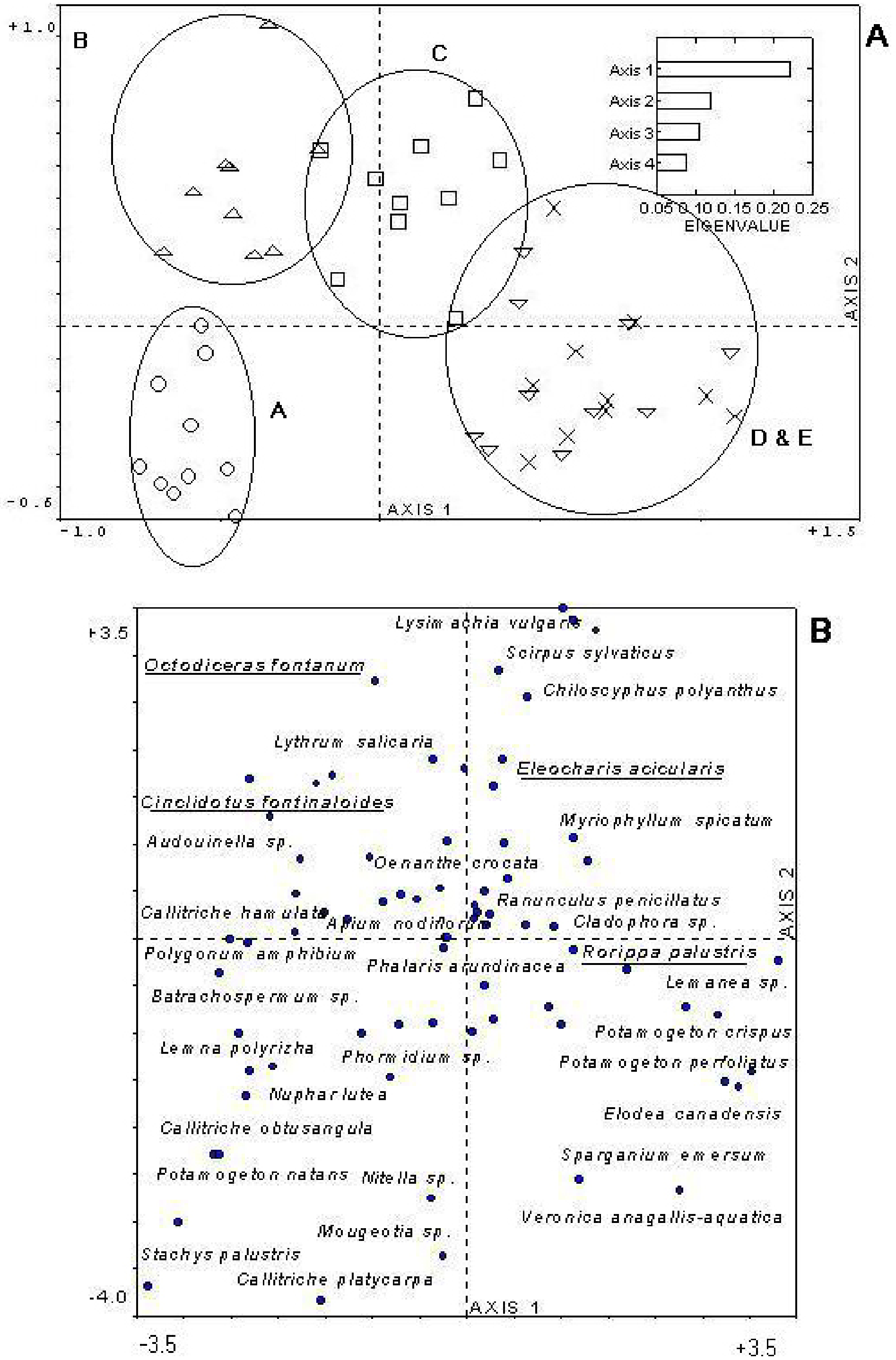

**FIGURE 3.** Correspondence analysis, distribution of (A) sites according to their floristic composition and (B) the principal aquatic plants of the hydro-peaking channel, on the plan formed by the first two axis, with respective eigenvalues of 0.22 and 0.12. Legend for symbols in part (A): circles stretch A, <0.1 km; point-up triangles stretch B, 0.1 to 1 km; squares stretch C, 1 to 5 km; point-down triangles stretch D, 5 to 10 km; X-crosses stretch E, 10 to 15 km. In (B) underlined species were specific for the site in the data of the 19th century (see Table 2).

The first axis (17.6% of the information) may be interpreted as a gradient of distance from the stretches in the immediate vicinity (A and B: <1 km) to the stretches further downstream (>5 km). In terms of species composition, it opposed the well-anchored and/or rheophilic taxa (*Nuphar lutea*, *Potamogeton natans*, *Polygonum amphibium*, *Callitriche* spp., aquatic bryophytes) species from the reservoir (*Lemna* spp.) and opportunistic macro-algae, to the unrooted or weakly-rooted hydrophytes (*Elodea canadensis*, *Myriophyllum spicatum*), pool hydrophytes, or species preferring clay substrata (*Potamogeton crispus*, *P. perfoliatus*) and emerged rhizophytes (*Sparganium emersum*, *Veronica anagallis-aquatica*).

The second axis (9.8% of the information) opposed the nearest stretches (<0.1 km) and those more downstream (>5 km) from the middle reaches (between 0.1 to 5 km). It may be related to the disturbance effects of the channelling that short-cuts the former channel (Fig. 1), where the middle stretches (1 to 5 km) presented more deposits[26] that braided the river bed (located in the positive part of the axis), and the stretches free of deposits situated very near or very far from the dam (in the negative part). In term of species composition, it opposed pure hydrophytes (*Nuphar lutea*, *Potamogeton natans*, *P. crispus*, *P. perfoliatus*, *Polygonum amphibium*, *Callitriche* spp., *Elodea canadensis*) to emerged rhizophytes or hygrophytes that colonised the deposits (*Lysimachia vulgaris*, *Lythrum salicaria*, *Eleocharis acicularis*, *Lycopus europaeus*), and bryophytes (*Octodiceras fontanum*, *Cinclidotus fontinaloides*, *Chiloscyphus polyanthus*) of the fast-flowing braided channel (the current acceleration was due to the extension of deposits and the reduction of the wetted bed[26]). However, there was a less marked difference here, between these free-flowing sections and the braided riverbed, than that assessed with the distance gradient in the first axis.

### Downstream Effects on Species Richness

Vegetation response to channelling and hydro-peaking was also analysed through the observed values of the richness of species of spermatophytes and bryophytes and genera of macroalgae (Fig. 4). The total number of taxa found was higher very near the dam in stretch A (<0.1 km), and values were found to be similar for the other stretches (B to E); the Wilcoxon test was used. Results of the statistical analysis are summarised in Table 1.

With 89 taxa present in the hydro-peaking channel downstream from the dam, stretch A presented the highest score, with 52 taxa and the highest richness per section (32), and with the lowest standard deviation (5). However, the other regulated stretches also showed a high richness with 38 to 44 taxa, and a mean per section of 19 to 25.

### Eco-Historical Approach

As regards the river, the spermatophyte results showed that in 1866, Mabille[23] identified an important specificity for this short river section (12 aquatic taxa on 101) before the dam was built, transformed to the present hydro-peaking channel. This was confirmed by Camus' works on aquatic bryophytes between 1876 and 1904[25], with 4 specific taxa in the hydro-peaking channel on 114 aquatic bryophytes for that regional scale study. Table 2 shows the list of specific taxa, six of which were not found in our study.

## DISCUSSION

Hydro-peaking events were moderate during the period of vegetative growth, suggesting that the gain in richness of the regulated reaches can be connected to the Intermediate Disturbance

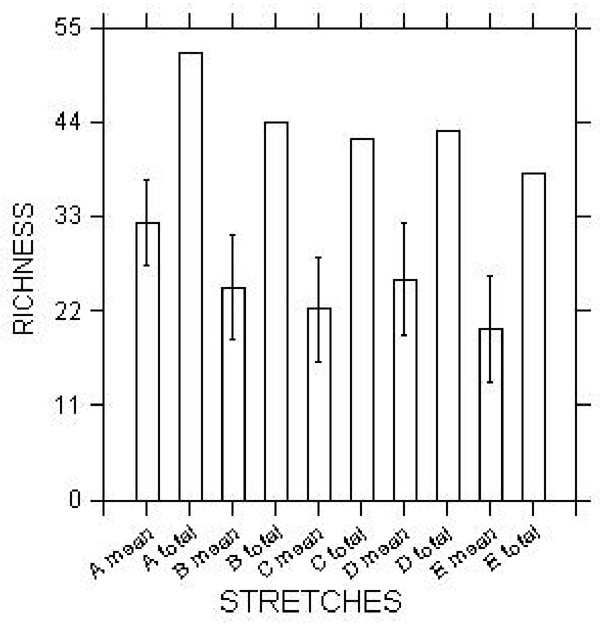

**FIGURE 4.** The mean richness of aquatic taxa on the 50-m stations and standard deviation during 1995 and 1996 (n = 10 in each case) and the total richness at the stretch scale downstream from the dam (A: <0.1 km; B: 0.1 to 1 km; C: 1 to 5 km; D: 5 to 10 km; E: 10 to 15 km).

### TABLE 1
## Comparison of the Floristic Lists of the Five Stretches A to E by Using the Nonparametric Paired Wilcoxon-Test

| | A | B | C | D | E |
|---|---|---|---|---|---|
| **A** | 1,00 | | | | |
| **B** | **0,06** | 1,00 | | | |
| **C** | **0,06** | 0,68 | 1,00 | | |
| **D** | **0,10** | 0,86 | 0,83 | 1,00 | |
| **E** | **0,01** | 0,26 | 0,39 | 0,23 | 1,00 |

*Note*: in bold are given the probabilities <10% of error of accepting a difference between two stretches.

### TABLE 2
## List of Aquatic Spermatophytes and Bryophytes Specific of the Site before the Dam Construction in the Sector of the Actual Channel[23,25]

**Spermatophytes specific of the site in 1866**

*Potamogeton gramineus* var *heterophyllus*
*Potamogeton pusillus (= P. panormitanus)*
*Carex acutiformis*
*Myosoton aquaticum*
*Rorippa palustris*
*Typha angustifolia*
*Eleocharis acicularis*
*Cyperus longus*
*Ranunculus trichophyllus**
*Senecio aquaticus* ssp *barbareifolius**
*Senecio aquaticus* ssp *aquaticus**
*Zannichellia palustris**

**Bryophytes specific of the site in 1866-1904**

*Cinclidotus tontinaloides*
*Fissidens julianus (Octodiceras fortanum)*
*Hygroam blystegium irriguum (Am blystegium tenax)**
*Drepanocladus aduncus gr pseudofluitans**

*Note*: Asterisks (*) indicate taxa that were not found in 1995–1996 in our study.

Hypothesis[26]. This model has been described for macroinvertebrate communities and extended to other general traits (physical or biological) of the downstream reaches of large regulated systems[3]. For aquatic plants, the Intermediate Disturbance Hypothesis has been used to explain the structure of the periphyton communities downstream from hydroelectric dams in the Durance river, in southern France[9], where different levels of discharge were compared. The global gain in richness throughout the hydro-peaking channel[17] must be carefully interpreted and detailed: inside the 15-km length of the channel, the aquatic macrophyte richness was high when compared with the unregulated reaches of the Rance[17,26] and with the other rivers of the region [33,34], of France [18,35], and of northern Europe [1,36]. In detail, the first stretch (A), close to the dam, presented more richness than the remaining channel, which also presented a high level of richness. This first stretch, situated upstream of the geological change and with high exposure to rapid water flow variations, could be related to the Intermediate Disturbance Hypothesis, considering the existing low level of discharge (in terms of both frequency and magnitude) during the vegetative growth period. The secondary consequence of the hydro-peaking events was to promote emerged rhizophytes, increasing the richness of the river due to deposit accumulation in the middle stretches, a phenomenon commonly observed in regulation works[37]. However, the richness of the channel overall had to be interpreted in relation to the eco-historical comparison results.

The fact that before the dam was built the specificity of the downstream channel was described for two of the principal taxonomic groups of aquatic plants by two different reference sources indicates how singular the site is from a floral point of view. The geology of the site, suddenly changing from siliceous to calcareous substratum, may lead to a potentiality of mixed flora at the interface. As dams are very often built on geological disruption[38], it is important to take the geological factors into account in such cases, in order to explain the composition of the vegetation[39,40]. The origin of the present flora is connected to the history of the river basin, as suggested by Nilsson for Nordic rivers: similar mechanisms for the richness of the vegetation along river banks between near stream systems of different size were due to the postglacial history of the rivers and of the surrounding landscape[4]; river basins did not always have the present degree of isolation[41]. In addition, a specific flora for terrestrial plants is found in the calcareous area near the river Rance[42].

The fact that most of the specific plants mentioned by the 19$^{th}$ century authors were also found in our study leads us to draw several inferences:

1. Aquatic plants have a high capacity to resist flood events, whether natural or not, and their recovery potential is high given this long-term pattern and comparison[43]. This is also suggested by the study of some biological traits of the different taxa. For stretch A, well-anchored taxa (*Nuphar lutea*, *Potamogeton natans*, gathered by Arber[41] in her plant traits classification), or hydrophytes with leaves that offer minimal resistance to the flow (*Callitriche* spp, *Ranunculus penicillatus*), or opportunistic macroalgae that could develop during the summer period between hydro-peaking events were selected. For the more downstream stretches (D, E) unrooted or weakly rooted plants such as *Elodea canadensis* or *Myriophyllum spicatum* were selected. However, the floral loss from the regulated channel could not only be explained by this flood influence hypothesis. For example, *Zannichellia palustris* is a plant with a potential rheophilic status, it was seen downstream from dams[44] and its absence leads us to infer that other processes could interfere with the physical process, e.g., competition processes between plants[45].
2. Hydraulic perturbations occurred before the dam construction: 42 sites of old water mills were counted over 67 km of the Rance, without considering its tributaries[26]. There were more than ten water mill sites in the inundated reservoir valley. In terms of aquatic plant selection, this leads us to question whether a single large hydro-electric power plant could have similar effects to those of several small water mills.

### CONCLUSIONS

The effects on the river plant communities downstream of the dam were significant, selecting plants along a distance gradient from the power station. A secondary effect was the role played by deposits. All these phenomena could explain the increasing richness in the hydro-peaking channel, a phenomena permitted by the low level of hydro-peaking events, in term of frequency and magnitude, during the vegetative growth periods. In some river sections, we could relate the increase to the Intermediate Disturbance Hypothesis; we explained the aquatic plant distribution by selection of various taxa as as result of particular physical perturbations that allowed their settlement or their maintain. The prevalent richness was also related to the specific singularity of the site. The fact that the dam is built on a geological disruption may contribute to floral diversity.

The river basin history cannot be considered from a purely geological perspective; more recently, the man-related impact was present before the dam was built, as industrialisation of the river was already underway in the 19th century. Water mill constructions and utilisations, and their consequences (human activities), may have heavily influenced the composition of the flora, and there should be careful interpretation of actual human effects, even if some questions will be difficult to resolve.

### ACKNOWLEDGEMENTS

Thanks to E.D.F. and the French Water Agencies for the financial support of the program, to Laurent Beillard for some figures, and Daniel Chicouène for his help in finding historical references.

### BIOSKETCH

**Ivan Bernez** is a Post-doctoral Fellow in the Forestry Department at the University of Lisbon. He earned his Ph.D. from the UMR Ecobiologie et Qualité des Hydrosystèmes continentaux of the Agronomic School of Rennes. His research interests include studying the ecological water quality in several countries (France, Hungary, French Polynesia and Portugal) since 1992, the specialization done during the Ph.D. (1995-99) on river macrophytes communities responses to chemical and hydraulic perturbations in Western France, followed by a post-doctoral period (2000-02) in South Portugal enlarging the themes of the Ph.D. with the study of aquatic plants invasions and riparian communities ecology, including biological and taxonomic aspects. University teaching on the domains has also been done since 1995 in France and Portugal.
